# An Optical Urate Biosensor Based on Urate Oxidase and Long-Lifetime Metalloporphyrins

**DOI:** 10.3390/s20040959

**Published:** 2020-02-11

**Authors:** Tokunbo Falohun, Michael J. McShane

**Affiliations:** 1 Department of Biomedical Engineering, 5045 Emerging Technologies Building, 3120 TAMU, Texas A&M University, College Station, TX 77843, USA; tfalohun@tamu.edu; 2 Department of Materials Science and Engineering, 3003 TAMU, Texas A&M University, College Station, TX 77843, USA

**Keywords:** urate, gout, optical biosensors, metalloporphyrins, phosphorescence

## Abstract

Gout is a condition that affects over 8 million Americans. This condition is characterized by severe pain, and in more advanced cases, bone erosion and joint destruction. This study explores the fabrication and characterization of an optical, enzymatic urate biosensor for gout management, and the optimization of the biosensor response through the tuning of hydrogel matrix properties. Sensors were fabricated through the co-immobilization of oxygen-quenched phosphorescent probes with an oxidoreductase within a biocompatible copolymer hydrogel matrix. Characterization of the spectral properties and hydrogel swelling was conducted, as well as evaluation of the response sensitivity and long-term stability of the urate biosensor. The findings indicate that increased acrylamide concentration improved the biosensor response by yielding an increased sensitivity and reduced lower limit of detection. However, the repeatability and stability tests highlighted some possible areas of improvement, with a consistent response drift observed during repeatability testing and a reduction in response seen after long-term storage tests. Overall, this study demonstrates the potential of an on-demand, patient-friendly gout management tool, while paving the way for a future multi-analyte biosensor based on this sensing platform.

## 1. Introduction

Uric acid is the end-product of purine nucleotide catabolism in humans. The compound, which is normally excreted by the kidneys or gastrointestinal tract, is produced by the liver from both exogenous and endogenous purine sources. These sources include purine-rich foods such as red meat and seafood (exogenous) and from endogenous purines derived from cell death [1,2]. In physiological conditions, uric acid mostly exists as its deprotonated resonance hybrid, urate, and associates with sodium ions to form monosodium urate (MSU) salts [3].

Abnormal kidney function or purine metabolism can result in hyperuricemia, in which there is an overproduction or insufficient removal of the compound from the bloodstream. Insufficient removal is most often the underlying cause of hyperuricemia and is associated with 90% of all cases [4,5]. Central to the removal of urate from serum are the kidneys, which play a critical role in regulating the compound in the body through a balance of reabsorption and secretion using urate transporters. There are several transporters that serve unique roles in the regulation of serum urate. Some transporters involved in urate resorption include urate transporter 1 (URAT1) and GLUT9 (SLC2A9), a voltage-sensitive transporter. Conversely, ABCG271 and NPT1/NPT4 (SLC17A1, SLC17A3) both serve as secretory urate transporters [6]. Therefore, the presence of dysfunctional variants of the genes associated with these transporters can lead to a significant increase in the risk of developing hyperuricemia [7]. Genes associated with key urate transporters include SLC22A12, SLC2A9, and ABCG2. Each of these genes carries instructions necessary for the synthesis of the aforementioned urate transporters. [8].

While hyperuricemia is associated with several health conditions such as cardiac disease, renal dysfunction, leukemia, and Lesch Nyhan Syndrome, the most prevalent condition is gout – a form of inflammatory arthritis [3,9]. Affecting over 8 million Americans, gout is characterized by severe pain, and in more advanced chronic cases, bone erosion and joint destruction [10,11]. This inflammatory condition is triggered by MSU crystal deposits in the synovial spaces of the joints, which form when serum urate levels rise above their physiological solubility limit of 6.8 mg/dL. In fact, the identification of these crystals in the synovial fluid using polarized light microscopy is the definitive method of diagnosing gout. However, most physicians do not use this method and instead base their diagnosis on several clinical criteria such as the frequency, location, and severity of the immune response [12,13].

If left untreated, gout can progress into a chronic progressive condition and spread to multiple joints, where the accumulation of urate crystals leads to the development of tophi, or deposits of urate crystals, in the synovial spaces [4]. Gout can have a significant impact on quality of life by severely limiting patient mobility and the freedom to pursue daily activities. The societal and economic burden of the condition is also increasing. Current estimates put annual work-related expenses for gout patients at $1300, in addition to $3000 in healthcare-related costs [14,15].

To reduce the risk of gout attacks, patients must maintain low serum urate levels (<6 mg/dL) through diet and antihyperuricemic medication to allow for the dissolution of urate crystals [10]. However, monitoring this metric can be a burden, and most patients wait until the onset of a gout attack to seek therapy. Studies of elderly gout patients in the U.S. show that less than 20% of patients had serum urate measurements in a given year, and 40–80% of patients fail to achieve the desired target serum urate levels (<6 mg/dL) [16]. Current methods of urate detection primarily consist of laboratory tests involving high-performance liquid chromatography, colorimetry, UV absorption, and amperometric methods [17,18,19,20]. However, these techniques can be costly and time-consuming due to the required reagents, equipment, labor, and sample preparation steps. This is particularly troublesome for chronic gout patients who more frequently need to monitor the effect of diet and lifestyle habits on their urate fluctuations in order to avoid gout flares.

In principle, portable urate test kits similar to commercially available glucose monitors could eliminate the need for expensive reagents and resources. However, these devices require drawing blood samples and can reduce patient compliance due to the pain involved, particularly in cases where the condition affects the joints in the hand. Additionally, these handheld meters have largely failed to demonstrate enough accuracy and precision for mass use, and are yet to obtain approval by the FDA. Clearly, there is a need for a more user-friendly and reliable means for patients to measure their urate concentration more frequently to better manage their condition.

Optical biosensors present a desirable option for on-demand urate determination to address the issue of needing blood draws. We have recently reported on a photoluminescent oxygen-sensing hydrogel platform that has been demonstrated to function as an indirect indicator for oxidase substrates such as glucose and lactate [21,22]. Unlike amperometric biosensors that rely on electrodes and peroxidases, which increase biosensor susceptibility to interferents like ascorbic acid, glucose, and urea [23], peroxidase-free optical biosensors present a means of avoiding the interference effects from analytes present in biofluids. In this study, we present a reagentless urate biosensor for implant and ex-vivo applications based on oxygen-quenched phosphorescent probes co-immobilized with an oxidoreductase within a biocompatible copolymer hydrogel matrix. Uricase, or urate oxidase, specifically catalyzes the oxidation of urate to allantoin (C_4_H_6_N_4_O_3_), hydrogen peroxide (H_2_O_2_), and carbon dioxide (CO_2_) via the following reaction:

(1) $$Urate+O_{2}+H_{2}O\overset{Urate\;Oxidase}{\Rightarrow }Allantoin+H_{2}O_{2}$$

The use of optical biosensors for urate determination based on enzymatic oxygen depletion was first demonstrated by Schrenkhammer and Wolfbeis who developed an ex-vivo urate biosensor based on oxygen-sensitive particles, containing ruthenium and iridium, embedded in a polyurethane hydrogel immobilized with uricase [24]. The authors demonstrated the capability to detect urate changes in a 0–33.6 mg/dL range using measurements of fluorescence intensity.

In the past few decades, metalloporphyrins have emerged as attractive indicators for in vivo biosensing applications [25]. This is largely due to their photophysical properties being well-suited for conducting time-resolved measurements. Specifically, many of these compounds possess extraordinarily long phosphorescence lifetimes, relatively high quantum yields, large Stokes shifts, and the ability to operate at near-IR wavelengths, where absorption by water and hemoglobin is minimal [26].

This class of oxygen-sensitive long-lifetime metalloporphyrin is collisionally quenched in the presence of oxygen due to the non-radiative transfer of energy from the excited phosphor to oxygen through molecular interactions between oxygen and the phosphor [27]. Thus, in an oxygen-rich environment, there is greater oxygen quenching, resulting in lower phosphorescence lifetimes and intensities. Pioneered by Vinogradov et al. [28], the photophysical properties of these metalloporphyrins were further improved by Niedermair et al. through the addition of benzene rings to the peripheral benzo groups in the palladium(II) and platinum(II) porphyrin structures to create a bathochromic shift of the emission bands [29].

In this study, palladium (II) tetramethacrylated benzoporphyrin (BMAP), an oxygen-sensitive long-lifetime metalloporphyrin, was used as the optical signal transducer, along with urate oxidase, for the indirect detection of urate. The novelty of this system lies in the combination of the porphyrin dye and oxidoreductase enzyme within a hydrogel matrix to create an optically responsive urate hydrogel. This approach is advantageous because: (1) It can be used for implantation to eliminate the need for biofluid sampling, and (2) it simplifies the measurement process and eliminates the need for large, expensive instruments for urate determination. This system can be used to conduct transcutaneous optical interrogation using time-domain lifetime measurements, which are particularly effective for temporal separation of the long-lifetime emission from light scattering and autofluorescence emitted from the surrounding tissue [30].

It is noteworthy that the host material for the phosphor and enzyme plays a critical role in determining the bioresponse characteristics of the system, because of the multiple substrates and kinetics involved in the reaction. Therefore, developing an understanding of the tradeoffs between matrix hydrophilicity, swelling, oxygen and urate diffusion, and sensitivity to urate is critical to achieving the desired response over a relevant range of urate concentrations. Further, the matrix properties directly control diffusion of interfering species and may also impact enzyme stability as well. Therefore, through a systematic analysis of matrices comprising 2-hydroxyethyl methacrylate (HEMA) and acrylamide (AAm) copolymers, this study focuses on investigating the practical effects of hydrogel matrix materials on these important characteristics of the urate with the goal of expanding the oxygen sensing platform for monitoring urate in chronic gout patients.

## 2. Experimental Section

### 2.1. Materials

First, 2-hydroxyethyl methacrylate (HEMA) and tetra(ethylene glycol) methacrylate (TEGDMA) were purchased from Polysciences (Warrington, PA). Uric acid was obtained from USB Corporation (Cleveland, OH). In addition, 2,2-dimethoxy-2-phenyl-acetophenone (DMAP), ethylene glycol, ascorbic acid, urea, allantoin, acetaminophen, and creatinine were purchased from Sigma-Aldrich (St. Louis, MO, USA). Acrylamide (AAm) and dimethyl sulfoxide (DMSO) were obtained from VWR (Radnor, PA, USA), and 3β-D-glucose was purchased from Macron Fine Chemicals (Center Valley, PA, USA). Palladium (II) tetramethacrylated benzoporphyrin (BMAP or PdBP) was donated by Profusa, Inc. (San Francisco, CA, USA). Urate oxidase from recombinant *Escherichia coli* and glucose oxidase were acquired from BBI Solutions (Cardiff, UK). 

### 2.2. Instrumentation

Absorbance and emission measurements were performed using an Infinite 200 PRO 96-well plate reader (Tecan, Männedorf, Switzerland). Radical crosslinking was initiated using a Blak-Ray B-100SP UV lamp from UVP (Upland, CA, USA). Flow-through experiments were conducted using peristaltic pumps (L/S 7550 pump drive), pump heads (Easy Load 3), and precision tubing (L/S Norprene Tubing A60 G, L/S 13, 50 ft) purchased from MasterFlex (Gelsenkirchen, Germany). Oxygen concentrations were adjusted using mass flow controllers (Type 1179A General Purpose Mass-Flo Controller) and a pressure control unit (PR 4000 F) from MKS Instruments (Andover, MA, USA). Optical interrogation of hydrogel samples was conducted using custom optical readers, described in previous studies by our group [21,22]. Each reader contained a red LED (Lumileds LUXEON Rebel, *λ_ex_* = 630 nm) for excitation and silicon photomultiplier tubes (SiPMT, SensL) for emission detection. During oxygen and glucose response testing, samples were housed in a custom-designed Delrin flow cell capable of holding four hydrogel samples and four optical readers. Changes in oxygen concentration during the oxygen response tests were verified using an OX-500 oxygen microsensor and PA2000 picoammeter (Unisense, Aarhus, Denmark).

### 2.3. Hydrogel Selection

HEMA was chosen as the primary monomer for the biosensor matrix due to soft but tough mechanical properties, optical clarity, and biocompatibility [31]. However, the maximum equilibrium swelling ratio of polyHEMA is thermodynamically limited to only 40% [32]. To improve swelling, HEMA is often copolymerized with more hydrophilic materials, such as acrylamide (AAm) [33]. Both HEMA and AAm are used in a wide variety of biomedical applications including scaffolds for tissue engineering, drug delivery, contact lenses fabrication, and polymeric coatings for biomedical devices [34,35]. When copolymerized, the hydrogels retain many of the same desirable properties to HEMA [36,37], while allowing for easy tuning of hydrogel swelling.

In sensing applications, greater gel swelling increases the hydrogel mesh size and results in a less tortuous path for analyte diffusion through the hydrogel matrix. This leads to increased access of the substrate (urate) to the immobilized enzyme (urate oxidase), thus increasing the oxygen depletion and lifetime of nearby phosphors. Changes in diffusivity and biosensor sensitivity after copolymerization with AAm can be quite pronounced. Previous reports indicated an approximate 30-fold increase in glucose diffusivity and a corresponding 6-fold increase in sensitivity of a glucose biosensor when acrylamide was copolymerized with HEMA in a 50:50 molar ratio as compared to a pure HEMA homopolymer [36]. Although not quite as pronounced, the copolymerization of AAm with HEMA also increased the diffusion and sensitivity of lactate, with the 75:25 composition having roughly a 2-fold increase in lactate diffusion and sensitivity when compared to a HEMA homopolymer [22]. Despite the improvement in biosensor sensitivity after copolymerization with AAm, copolymers with a high content of AAm (≥50%) also demonstrated less consistent and repeatable behavior; this was attributed to the greater phase separation and inhomogeneous dispersion of the dye and enzyme [36]. Considering the tradeoffs in sensitivity and reproducibility, 90:10, 75:25, and 50:50 poly(HEMA-co-AAm) compositions were explored in this study for use in a urate biosensor.

### 2.4. Hydrogel Fabrication

All copolymer compositions of HEMA and AAm were fabricated using a modified version of a previously described method [22]. Specifically, a 250 µL precursor solution of HEMA and AAm was prepared in a microcentrifuge tube by combining the appropriate volume percentage of HEMA and a 67.2 v/v% solution of AAm dissolved in water (i.e., 125 µL of HEMA and 125 µL of the AAm solution for a 50:50 composition) and also adding 2.5 mg of DMAP. Next, 5 µL of TEGDMA was added to the precursor solution and vortexed. To enhance the homogeneity of the mixture, 90 µL of ethylene glycol co-solvent was mixed into the precursor solution and vortexed again. Afterward, 49 mg (250 units) of uricase dissolved in 107.5 µL of 10 mM phosphate-buffered saline (PBS), along with 10 µL of 10 mM BMAP dissolved in DMSO, was pipetted into the solution. The solution containing the enzyme and dye was further mixed to ensure homogenous dispersion. 

The precursor solution was pipetted into a hydrogel mold created using 2 glass slides separated by a 0.03’’ thick Teflon spacer for radical crosslinking under a UV lamp for 3 min on each side. After crosslinking, the newly formed hydrogel gel slab was extracted from the mold, rinsed with deionized water, stored in a 10 mM PBS (pH 7.4) solution, and refrigerated overnight at 4 °C. For all testing and characterization, samples were taken from the hydrogel slab using a 6 mm diameter circular biopsy punch (VWR, Randor, PA, USA).

### 2.5. Swelling Ratio

After hydrogel fabrication, three samples were stored in deionized water for 24 h at 25 °C to allow samples to reach their equilibrium swelling volume. To obtain the hydrated mass, *W_s_*, the samples were weighed using an analytical scale after blotting off excess water. Samples were then placed in a desiccator for 24 h prior to weighing their dry mass, *W_d_*. The swelling ratio is calculated using the following equation:

(2) $$Swelling\;Ratio\;=\;\frac{W_{s}-W_{d}}{W_{d}}\;\times \;100.$$

### 2.6. Oxygen Response

**Figure 1:**
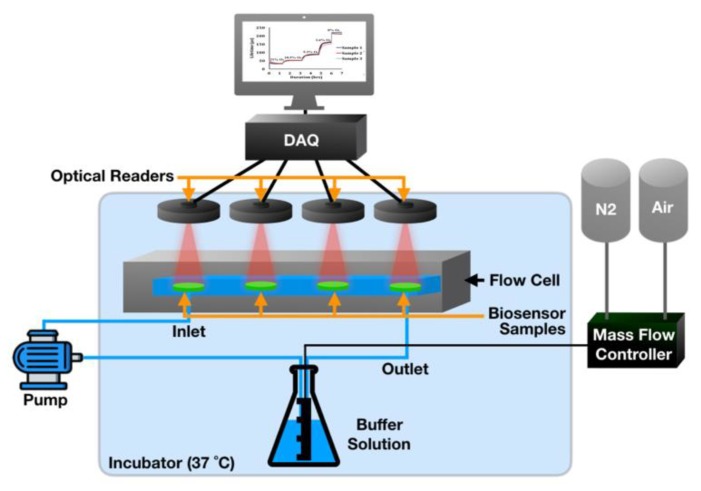
Schematic of flow-through system for in vitro biosensor assessments of oxygen response.

To assess oxygen response of the urate biosensors, 6 mm biosensor punches (n = 3) and a reference oxygen sensor were placed into a custom Delrin flow cell with a recirculating flow (4 ml/min) of 10 mM PBS (pH 7.4) enabled by a peristaltic pump connected to two little Erlenmeyer flasks containing the buffer solution. Using the mass flow controllers, custom ratios of nitrogen and air were bubbled into the reservoir to adjust the dissolved oxygen concentrations in a step-wise fashion to achieve 21%, 10.5%, 5.25%, and 2.1% dissolved oxygen in the PBS, as confirmed with the oxygen electrode. This flow-through system is depicted in Figure 1.

To achieve 0% oxygen, a chemically induced oxygen purge was performed by combining an eight-molar-glucose solution with a 30 µM glucose oxidase solution in a 3:1 volume ratio. The biosensor responses to known oxygen values were recorded by means of the custom lifetime measurement system. All experiments were carried out in incubators to ensure a constant temperature of 37 °C.

The oxygen-induced collisional quenching of the immobilized porphyrins in the host hydrogels is generally described mathematically by the Stern–Volmer relationship.

(3) $$\frac{\tau_{0}}{\tau }=1+K_{sv}\left[O_{2}\right],$$

where *τ* represents the luminescence lifetime at a particular oxygen concentration [$O_{2}$], and $\tau_{0}$ represents the lifetime in the absence of oxygen. The sensitivity of each copolymer composition to changes in oxygen was characterized by the Stern–Volmer constant (*K_sv_*).

### 2.7. Optical Measurements

Using the LabVIEW program, the LED contained in the optical reader was powered at 200 mA, pulsed on for 500 µs, and turned off for 2500 µs to allow for emission signal collection. The optical readers were mounted onto the flow cells containing the hydrogel samples. Phosphorescence lifetime (τ) was calculated using the LabVIEW program, which extracted the lifetime from the signal decay detected from the photomultiplier tubes by nonlinear least-squares curve fitting to a single exponential decay.

### 2.8. Urate Response

**Figure 2:**
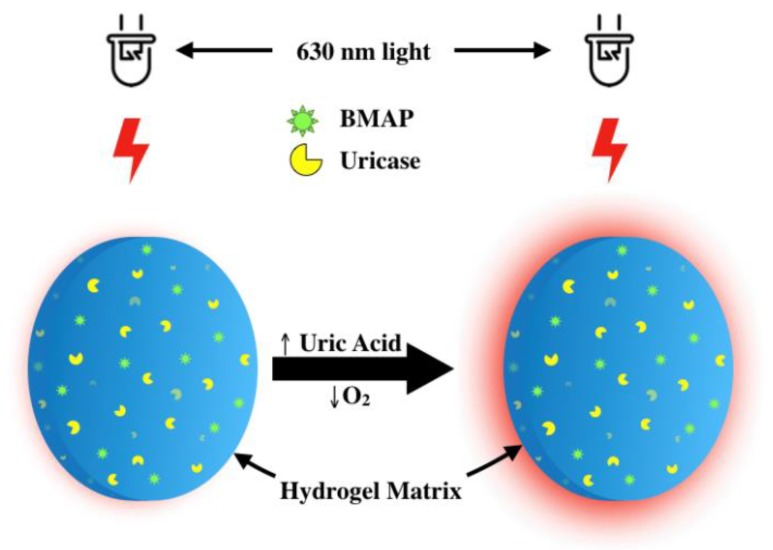
Illustration of the urate biosensing mechanism based on enzyme-induced oxygen depletion coupled with oxygen-responsive phosphors immobilized in a biocompatible hydrogel matrix. Urate concentration is proportional to phosphorescence intensity and lifetime.

In the enzymatic system described, urate concentrations are indirectly determined through changes in phosphorescence emission lifetime, which increases when there is a local depletion of oxygen by uricase in the presence of urate, as illustrated in Figure 2.

Urate response was assessed using a similar technique to the previously described oxygen response measurements. However, instead of changing oxygen concentration, the concentration of urate in solution was varied. This was achieved using peristaltic pumps, which were used to mix a 0 mg/dL urate solution (10 mM PBS) with a 10 mg/dL urate solution (dissolved in 10 mM PBS) to achieve a series of different concentrations within a 0–10 mg/dL analyte range. The mixed solution was fed to a flow cell containing the urate-responsive hydrogel samples and was output to a waste container. The lifetime was measured continuously and allowed to reach a steady state before changing the concentration (roughly 60 min for each concentration). 

The limit of detection was calculated as the uric concentration corresponding to the phosphorescence lifetime at 0 mg/dL urate plus three times the standard deviation of the lifetime signal at that analyte concentration. The sensitivity of the urate response, or slope of the calibration curve, was also calculated as a key figure of merit to characterize sensor response. The copolymer composition with the highest sensitivity and lowest limit of detection was used for all further evaluations.

### 2.9. Selectivity

Selectivity of the biosensor was evaluated by exposing biosensor samples to PBS containing physiologically relevant concentrations of select common analytes including ascorbic acid, glucose, urea, allantoin, acetaminophen, creatinine, and urate for one hour each, using the flow-through system. The luminescent measurement system was used for optical interrogation, and the corresponding percent change in phosphorescence lifetime was calculated for each analyte.

### 2.10. Storage Stability

To evaluate the long-term stability of the biosensors, samples were stored in two buffer conditions and assessed every 4 weeks over an 8 week total experimental test duration. The samples were tested under two storage conditions over this period. In the first condition, hydrogel samples were stored in PBS containing no urate at 23 °C. In the second condition, samples were stored in PBS containing 6.8 mg/dL urate at 23 °C. All solutions contained 10 mM PBS (pH 7.4). The percentage of the initial lifetime response retained over the 8-week duration was recorded.

## 3. Results and Discussion

### 3.1. Swelling Ratio

Changes in hydrogel swelling were investigated for each copolymer composition. As noted, acrylamide is known to be more hydrophilic than HEMA due to the presence of amine side groups in its structure. Conversely, the swelling of polyHEMA is limited by inter- and intra-molecular interactions. Specifically, intramolecular hydrogen between amide groups, along with inter- and intramolecular bonding between amide and hydroxyl groups in hydrogels containing high concentrations of HEMA, restricts gel swelling [38]. Therefore, adjusting the ratio of each monomer allows for the tuning of the swelling ratio of the polymer matrix.

As expected, increasing the acrylamide concentration resulted in a greater swelling ratio, as shown in Table 1. Differences in the degree of swelling between the hydrogel compositions were quite pronounced, with the 50:50 poly(HEMA-co-AAm) hydrogel composition having approximately 250% the swelling ratio of the 90:10 composition. On the other hand, the 75:25 composition had only a ~30% increase in swelling ratio, which implies that the swelling ratio is not directly proportional to acrylamide concentration. A similar difference between the swelling ratio of the 75:25 and 50:50 compositions was observed in previous studies of glucose biosensors fabricated using similar hydrogel systems [39]. In addition to the presence of amide side groups, the exponential increase in swelling upon the addition of acrylamide may also be due to the partial hydrolysis of the moiety, which has been observed in pHs of 6 and above. Specifically, the formation of negatively charged carboxylate ions following acrylamide hydrolysis can lead to greater elastrostatic repulsion within the gel and increased hydrogel swelling [40,41].

### 3.2. Absorbance and Emission Spectra of Urate Biosensors

**Figure 3:**
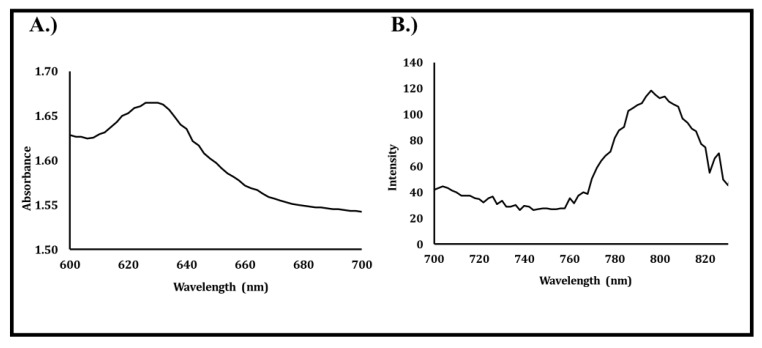
(**A**) Absorbance and (**B**) emission spectra of urate biosensor containing palladium (II) tetramethacrylated benzoporphyrin (BMAP) and uricase.

Absorption and emission spectra of urate biosensors composed of 50:50 poly(HEMA-co-AAm) containing BMAP and uricase were measured and are depicted in Figure 3A,B. The “optical window,” which occurs at wavelengths falling between 600 and 950 nm, must be considered when selecting probes for transdermal interrogation [25,42]. At wavelengths shorter than 600 nm, the strong absorption, scattering, and autofluorescence of light by pigments like hemoglobin found in tissue present a challenge to conducting transdermal optical interrogations. On the other hand, at wavelengths longer than 950 nm, light absorption by water presents a similar challenge. As such, near-IR probes, such as BMAP, with excitation and emission bands that fall within the “optical window” are desirable for use in implantation applications. 

In this system, an absorption peak was observed around 633 nm, which would allow for deep tissue penetration and optical interrogation of hydrogel implants, as the emission peak was also detected around 795 nm, well within the “optical window” where scattering and absorbance from tissue chromophores are relatively low and absorption by water is insignificant.

### 3.3. Effect of Oxygen Concentration on Biosensor Response

**Figure 4:**
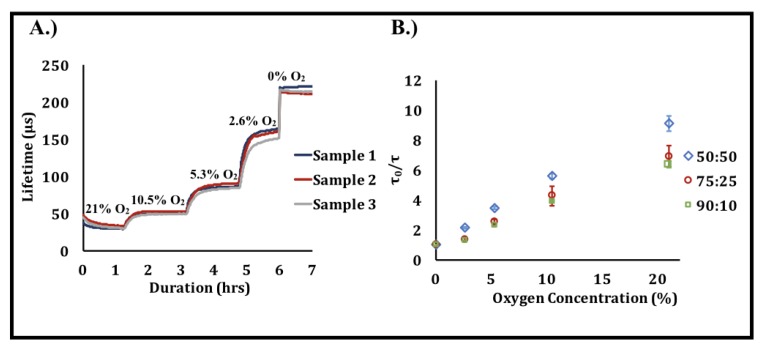
Effect of oxygen concentration on phosphorescence lifetime. (**A**) Raw data illustrating the changes in phosphorescence lifetime over time as oxygen concentration is reduced [n = 3 samples, 50:50 poly(2-hydroxyethyl methacrylate (HEMA)-co-acrylamide (AAm))]. These data are representative of a typical oxygen response profile obtained from a single oxygen response test. (**B**) Oxygen response of the three hydrogel urate biosensor compositions. Each data point represents a steady-state average of n = 3 samples of the sample composition. Error bars represent 95% confidence intervals.

Figure 4 shows the response to changes in phosphorescence lifetime as a function of oxygen concentration in the three hydrogel compositions as a result of the oxygen-induced collisional quenching of the immobilized porphyrins. The sensitivity of each copolymer composition to changes in oxygen was characterized by the Stern–Volmer constant (*K_sv_*), which corresponds to the slope of the Stern–Volmer plot (Figure 4); greater *K_sv_* values correspond to higher sensitivities.

The *K_sv_* values shown in Table 2 indicate that the difference between the hydrogel compositions was insignificant, which closely aligns with findings in prior studies [22]. As oxygen has a very small molecular size and is likely not significantly affected by the difference in hydrogel mesh size, this finding is unsurprising. Based on these observations, we can infer that the hydrogel matrix likely does not serve as a significant diffusion barrier to oxygen; hence, changes in the copolymer composition within a 50:50–90:10 poly(HEMA-co-AAm) range likely does not affect the ability of oxygen to access the immobilized porphyrin.

### 3.4. Effect of Urate on Phosphorescence Lifetime

**Figure 5:**
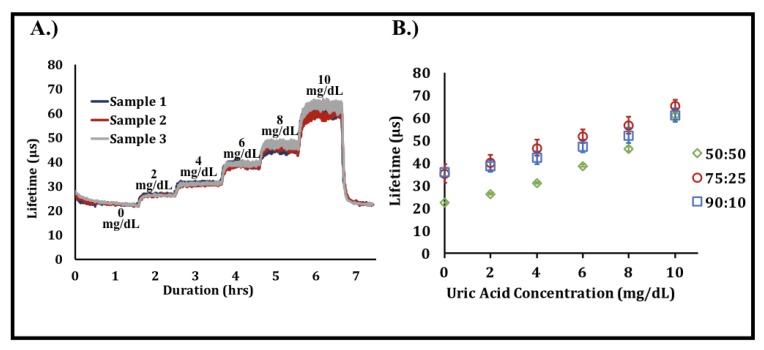
Effect of urate concentration on phosphorescence lifetime. (**A**) Raw data illustrating the change in phosphorescence lifetime over time with increases in urate concentration. Data are representative of a typical urate response profile obtained from a single test [n = 3 samples, 50:50 poly(HEMA-co-AAm)]. (**B**) Urate responses obtained from the three different hydrogel urate biosensor compositions. Each data point represents a steady-state average of n = 3 samples of the sample composition. Error bars represent 95% confidence intervals.

The effect of urate concentration on phosphorescence lifetime was investigated over a 0–10 mg/dL analyte range. Normal physiological concentrations of urate in human serum range from 2.5 to 7.5 mg/dL, with concentrations above 6.8 mg/dL generally considered as hyperuricemic [43]. As shown in Figure 5, all sensors demonstrated a monotonic, highly linear increase in phosphorescence lifetime with increased urate concentrations. This is a result of the local oxygen depletion created by uricase in the presence of urate, which leads to a reduction in the collisional quenching of the phosphor by oxygen and greater luminescence lifetimes. Response times for the sensors were roughly 15–20 min and are illustrated by the duration necessary to achieve a steady-state phosphorescence lifetime after changes in urate concentration were made.

Increasing the hydrogel swelling ratio through acrylamide copolymerization was expected to improve the urate biosensor sensitivity, as it was hypothesized that urate would more freely diffuse into the hydrogel matrix to drive enzymatic oxygen depletion. The results presented herein match that expectation, as sensors with higher concentrations of acrylamide produced greater sensitivities (Table 3). As shown in Figure 5B, the 50:50 composition possessed lower phosphorescence lifetimes than the other compositions, even with no urate present. This observation implies that, although oxygen diffuses through the three matrices at similar rates, as shown in the oxygen response tests, there is likely greater oxygen access to the immobilized phosphors in the 50:50 compositions when compared to the other two compositions. This phenomenon is likely driven by the increased swelling present in this composition due to its higher acrylamide content.

What is important to note about this point is that the three selected copolymer compositions possess diffusivity properties that are well-suited for the described sensing system, while also maintaining low variation between samples. Prior glucose diffusion studies suggest that pure HEMA-based sensors possess much lower analyte diffusivity values (by two orders of magnitude) and, thus, lower sensitives than HEMA-AAm copolymers [36]. Despite the structural difference in glucose and urate, we expect to observe a similar reduction in diffusion of both small molecules, as both molecules have similar molecular weights (glucose = 180.156 g/mol, urate = 168.112 g/mol). However, such a reduction in analyte diffusion/sensitivity would be particularly problematic in this urate sensing system due to the physiological concentration of urate, which is roughly two orders of magnitude lower than that of glucose [1,44].

On the other hand, too much acrylamide was found to lead to greater phase separation and inhomogeneous dispersion of the dye and enzyme in prior studies. This resulted in increased variation between samples [22,36]. Interestingly, this increased sample variation with higher acrylamide concentrations was not observed in the presented urate biosensors. The acrylamide-induced phase separation seen in previous cases may be offset by the amphiphilic nature of uricase, which is present in greater amounts in this system to compensate for its relatively low activity. Specifically, while uricase readily dissolves in water, it contains nonspecific hydrophobic binding sites in its protein structure that likely improves the solubility of BMAP in the hydrogel matrix. Improper mixing of the enzyme and dye into the hydrogel matrix is another possible source of sample variation. This is particularly true when the enzymes aggregate or the hydrophobic dye precipitates into clusters, which may prevent even oxygen access and quenching.

Despite the capability to detect urate changes in a physiologically relevant range, the sensitivity of the biosensors can be further improved. The peak lifetime of BMAP in a 50:50 poly(HEMA-co-AAm) urate biosensor is roughly 240 µs in the absence of oxygen, while the peak lifetime of the same sensor in a 10 mg/dL urate solution only reaches 65 µs. As changes in urate concentration are resolved through changes in phosphorescence lifetime, this indicates that the biosensor sensitivity is much lower than the system is capable of producing. The low peak lifetime is likely due to the relatively low levels of urate tested, coupled with the low activity of uricase relative to oxidoreductases like glucose oxidase. Increasing the lifetime range will increase the biosensor resolution. Possible methods to achieve this include using a hydrogel matrix with a greater mesh size, or increasing enzyme concentration and bioactivity though chemical or genetic modification. It is also important to appreciate that this lower sensitivity when tested under ambient conditions is preferred when using the sensors in low-oxygen environments such as those found in biological tissue, which will be investigated in future experiments.

### 3.5. Selectivity

The effect of various organic metabolites found in human serum on the phosphorescent signal was investigated to assess the selectivity of the urate biosensor system. Due to the use of urate oxidase, a high degree of urate selectivity was expected, with negligible interference from other species. As shown in Table 4, the system was indeed most sensitive to urate. However, when the biosensor samples were exposed to glucose and sucrose, responses equivalent to 10.89% and 17.77% of the urate response were observed, respectively. As neither analyte is known to be catalyzed by or interfere with uricase [45,46,47], these unexpected responses produced by glucose and sucrose could be generated by an analyte-induced alteration of the hydrogel environment. Alternatively, the dissolution of the glucose and sucrose in the buffer solution may have altered the concentration of dissolved oxygen, leading to an increase in phosphorescent lifetime. This phenomenon has been observed after the dissolution of glucose in culture medium [48]. Regardless, further investigation needs to be conducted to explore the effect of glucose and sucrose on the urate biosensor. All other analytes showed less than 5% of the urate response. Each analyte was tested at approximately physiological concentrations in PBS buffer, pH 7.4. 

Considering the relatively low concentration of urate relative to metabolites like glucose and urea, a urate biosensor must be highly specific when exposed to such compounds. Hence, semipermeable membranes are typically employed for amperometric biosensors [49,50]. A key advantage of this sensing approach is the selectivity granted by the use of a highly specific enzyme coupled with a signal transduction method unaffected by reducing agents like ascorbic acid. On the other hand, local changes in oxygen concentration can affect sensing accuracy due to the oxygen dependence of the system. To combat this issue for in vivo applications, an enzyme-free sample can be used as an oxygen reference to compensate for such changes in oxygen supply.

### 3.6. Storage Stability Results

**Figure 6:**
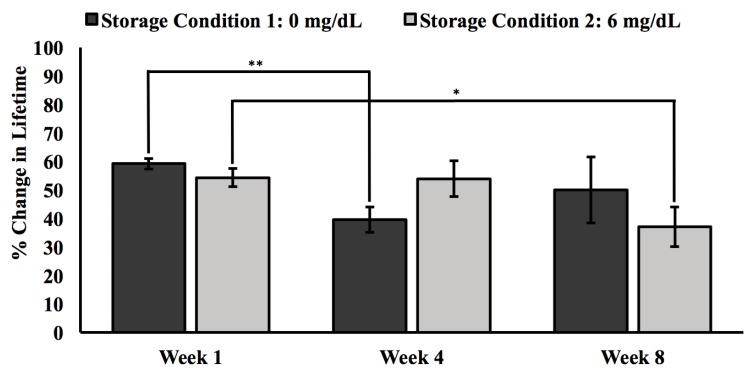
Response of urate biosensors stored in 5 mg/dL urate solution dissolved in 10 mM PBS. Both solutions were stored at 25 °C and pH 7.4 over an eight-week period. Measurements were made at the beginning, middle, and end points of the eight-week period. Error bars represent 95% confidence intervals. A single asterisk indicates a p value of 0.05 or less, while a double asterisk denotes a p value of 0.01 or less.

In addition to enabling the tuning of analyte diffusion and serving as a biocompatible interface, the poly(HEMA-co-AAm) matrix physically immobilizes the enzyme and chemically crosslinks with the terminal vinyl group of BMAP. Such hydrogel immobilization techniques have been shown to preserve the bioactivity of enzymes and long-term biosensor performance [51,52]. Physical entrapment of the enzyme within the polymer mesh of the hydrogel matrix reduces bioactivity loss due to changes in protein conformation, often seen in covalent enzyme immobilization techniques [53,54]. However, physical immobilization without formation of chemical bonds to anchor the enzyme to the hydrogel matrix carries the risk of enzyme leaching. For this reason, shelf-life tests were conducted to evaluate the performance of the urate biosensors over an eight-week period in 10 mM PBS solutions of 0 mg/dL (Condition 1) and 6 mg/dL urate (Condition 2), as shown in Figure 6.

After eight weeks of storage, sensors stored in Condition 1 retained 85% of their initial response, while the sensors stored in Condition 2 retained 68% of their initial response. This decrease in biosensor sensitivity seen in sensors in both storage conditions is mostly likely a result of enzyme bioactivity loss and leaching over time. The greater loss observed in sensors stored in 6 mg/dL urate may be attributed to enzyme degradation triggered by hydrogen peroxide produced during urate catalysis while the sensors were in storage [55].

However, an unexpected trend was observed in samples stored in Condition 1. After four weeks in storage, samples showed a steep decrease (33%) in percent change in lifetime and a 21% increase in response at the eight-week time point. This trend implies that sensors lost sensitivity after four weeks but regained function after eight weeks. Considering the magnitude of change in sensor response after four weeks and the irreversible nature of enzyme degradation, this anomaly is likely an experimental artifact. Unexpected decreases in phosphorescent lifetime can be a result of improper mixing creating hydrogel regions/samples with less available uricase to drive oxygen depletion. On the other hand, the formation of dye clusters after improper mixing of the hydrogel precursor solution can create an artificial increase in phosphorescent lifetime, as oxygen fails to access the core of the dye cluster. Overall, this urate biosensing system represents an early iteration of a unique approach that would allow for the long-term measurement of uric without blood draws using a hydrogel implant that is completely free from electronics and the need of a power source.

## 4. Conclusions

This study describes a simple, reusable, highly selective optical urate biosensor based on the co-immobilization of urate oxidase and oxygen-sensitive phosphors within a biocompatible hydrogel matrix. With use of long-lifetime benzoporphyrin molecules for optical signal transduction, the described system is especially suited for implantation because of the ability to transdermally interrogate the sensors without significant signal attenuation from scattering, absorption, and background autofluorescence from the local tissue environment. The sensors exhibited a linear relationship between phosphorescence lifetime and urate concentration over a range of 0–10 mg/dL urate. Hydrogel compositions of greater AAm concentrations were found to be more sensitive to urate, making the 50:50 poly(HEMA-co-AAm) composition the preferred choice for urate sensing applications. The system also demonstrates good selectivity and stability to changes in urate concentration in physiological conditions. While this study characterized the in vitro performance of the urate biosensing platform, future research efforts should investigate methods to improve the long-term stability of the system and optimize the sensors for in vivo conditions that will allow translation of this technology to real-world applications.

It is noteworthy that this application leverages a platform of oxygen sensors used with enzymes, as was previously shown for glucose and lactate sensing; with the use of alternate oxidoreductase enzymes, the same sensing technique can be used for the detection of other analytes or potentially in a multianalyte biosensor. For practical use as implantable biosensors for gout management, further studies should be conducted to increase further biosensor sensitivity, calibrate for local changes in oxygen concentration, and further investigate the relationship between interstitial and serum levels of urate [56,57].

## Figures and Tables

**Table 1:** Swelling ratios of urate biosensors.

Hydrogel Composition	Swelling Ratio
50:50 poly(HEMA-co-AAm)	249 ± 9.81^a^
75:25 poly(HEMA-co-AAm)	131 ± 4.32^b^
90:10 poly(HEMA-co-AAm)	100 ± 6.19^c^

Note: Different letters indicate significance in statistical difference between the mean swelling ratios of the compositions. Confirmed using one-way analysis of variance (ANOVA) using an ∝ of 0.05.

**Table 2:** Stern–Volmer constants of three urate biosensor compositions. Each value is an average of three samples ±95% confidence intervals.

Composition	*K_sv_* (% O_2_)^–1^
50:50 poly(HEMA-co-AAm)	0.32 ± 0.06
75:25 poly(HEMA-co-AAm)	0.29 ± 0.02
90:10 poly(HEMA-co-AAm)	0.27 ± 0.01

Note: Lack of significance in the difference between the mean *K_sv_* of the compositions was confirmed using a one-way ANOVA using an ∝ of 0.05.

**Table 3:** Key metrics for biosensor compositions. Each value is an average of three samples ±95% confidence intervals.

Composition	LOD (mg/dL)	Sensitivity µs/(mg/dL)
50:50 poly(HEMA-co-AAm)	2.28 ± 1.23	3.71 ± 0.17
75:25 poly(HEMA-co-AAm)	4.01 ± 1.18	2.92 ± 0.36
90:10 poly(HEMA-co-AAm)	3.13 ± 0.72	2.46 ± 0.09

**Table 4:** Selectivity of urate biosensor.

	Concentration (mg/dL)	Percent Change
Urate	5	28.92 ± 2.10
Ascorbic Acid	1.8	1.17 ± 0.19
Glucose	90	3.15 ± 2.29
Sucrose	2.7	5.14 ± 3.25
Fructose	0.15	0.99 ± 0.61
Urea	36	0.45 ± 0.13
Allantoin	0.25	1.14 ± 0.61
Creatine	2.6	0.73 ± 0.51
Creatinine	0.9	0.63 ± 0.33
Acetaminophen	0.9	0.48 ± 0.16
